# Incorporation of *Actinobacillus pleuropneumoniae* in Preformed Biofilms by *Escherichia coli* Isolated From Drinking Water of Swine Farms

**DOI:** 10.3389/fvets.2018.00184

**Published:** 2018-08-14

**Authors:** Flor Y. Ramírez-Castillo, Abraham Loera-Muro, Nicy D. Vargas-Padilla, Adriana C. Moreno-Flores, Francisco J. Avelar-González, Josée Harel, Mario Jacques, Ricardo Oropeza, Carolina C. Barajas-García, Alma L. Guerrero-Barrera

**Affiliations:** ^1^Departamento de Morfología, Centro de Ciencias Básicas, Universidad Autónoma de Aguascalientes, Aguascalientes, Mexico; ^2^CONACYT, Centro de Investigaciones Biológicas del Noreste (CIBNOR), La Paz, Mexico; ^3^Departamento de Fisiología y Farmacología, Centro de Ciencias Básicas, Universidad Autónoma de Aguascalientes, Aguascalientes, Mexico; ^4^Groupe de Recherche sur la Maladies Infectieuses en Production Animale (GREMIP), Faculté de Médecine Vétérinaire, Université de Montréal, St-Hyacinthe, QC, Canada; ^5^Departamento de Microbiología Molecular, Instituto de Biotecnología, Universidad Nacional Autónoma de México, Cuernavaca, Mexico

**Keywords:** *Actinobacillus pleuropneumoniae*, *Escherichia coli*, respiratory pathogen, pleuropneumonia, biofilms, drinking water

## Abstract

*Actinobacillus pleuropneumoniae*, the etiological agent of porcine pleuropneumonia, represents one of the most important health problems in the swine industry worldwide and it is included in the porcine respiratory disease complex. One of the bacterial survival strategies is biofilm formation, which are bacterial communities embedded in an extracellular matrix that could be attached to a living or an inert surface. Until recently, *A. pleuropneumoniae* was considered to be an obligate pathogen. However, recent studies have shown that *A. pleuropneumoniae* is present in farm drinking water. In this study, the drinking water microbial communities of Aguascalientes (Mexico) swine farms were analyzed, where the most frequent isolated bacterium was *Escherichia coli*. Biofilm formation was tested *in vitro*; producing *E. coli* biofilms under optimal growth conditions; subsequently, *A. pleuropneumoniae* serotype 1 (strains 4074 and 719) was incorporated to these biofilms. Interaction between both bacteria was evidenced, producing an increase in biofilm formation. Extracellular matrix composition of two-species biofilms was also characterized using fluorescent markers and enzyme treatments. In conclusion, results confirm that *A. pleuropneumoniae* is capable of integrates into biofilms formed by environmental bacteria, indicative of a possible survival strategy in the environment and a mechanism for disease dispersion.

## Introduction

*Actinobacillus pleuropneumoniae* is a Gram-negative coccobacillus, pleomorphic, facultative anaerobe, non-spore forming, encapsulated (1) and a member of the *Pasteurellaceae* family (2–4). *A. pleuropneumoniae* is the etiological agent of porcine pleuropneumonia; one of the most important health problems in the swine industry worldwide, and along with other porcine respiratory pathogens, this pathogen is also included in the Porcine Respiratory Disease Complex (5–7). Isolates can be classified into two biotypes depending on their requirement for nicotinamide adenine dinucleotide (NAD-dependant and NAD-independant). There are 16 recognized serovars (8). In Mexico, swine pleuropneumonia is widespread (6, 7, 9–11). Infection usually occurs through air or by direct contact. The microorganism is able to colonize the tonsils and to adhere to the alveolar epithelium. In general, the initial step is the bacterial colonization and adhesion to host cells (12).

Biofilms are microorganisms three-dimensional complex communities embedded in an extracellular matrix, where displayed characteristic phenotypes are similar to the free-living organisms, also known as planktonic (13–18). Biofilms have a dynamic structure in which a multitude of metabolic interactions between neighboring cells are developed (19). Naturally, the dominant growth of microorganisms is through multi-species consortia, regulated by a variety of important intra- and inter-specific interactions in development, composition, structure, and function (20–22). These bacterial microbial communities constitute a multi-species society, with its own “rules and patterns of behavior” (23).

Recently, (24) described the involvement of biofilm formation during the infection process of *A. pleuropneumoniae*. However, few studies have been done on its ability to survive outside of the pig so as to be considered an obligate pathogen. Assavacheep and Rycroft (25) investigated the survival of *A. pleuropneumoniae* under controlled laboratory conditions. In aqueous suspension, survival was improved by the presence of NaCl and mucin; as well as lowered temperature. Our group has detected the presence of *A. pleuropneumoniae* in drinking water from pig farms in Mexico using antibodies and a specific PCR for the gene of the ApxIV toxin (6, 26). Subsequently, we evaluated the ability of *A. pleuropneumoniae* to form multi-species biofilms with other swine bacterial pathogens in the absence of pyridine compounds (nicotinamide mononucleotide [NMN], riboside nicotinamide [NR], or nicotinamide adenine dinucleotide [NAD]) that are essential for growth of *A. pleuropneumoniae* (27). *A. pleuropneumoniae* was able to grow with all species tested in the absence of pyridine compounds. Furthermore, *A. pleuropneumoniae* was able to form strong biofilms when mixed with *Streptococcus suis, Bordetella bronchiseptica*, or *Staphylococcus aureus*. Notably, in the presence of *Pasteurella multocida*, and *Escherichia coli, A. pleuropneumoniae* was able to form a two-species biofilm, although this was weaker than the biofilms formed with other bacteria (27).

In this study, *Escherichia coli* strains isolated from the microbial community of drinking water of swine farms of the State of Aguascalientes were characterized and evaluated to explore their possible interaction with *A. pleuropneumoniae* to form two-species biofilms, suggesting a possible mechanism used by *A. pleuropneumoniae* to survive in the drinking water in pig farms, and in the environment. Furthermore, changes in the composition of the extracellular matrix during the formation of these two-species biofilms were also characterized.

## Materials and methods

### Sampling of drinking water in swine farms

The study was performed in the swine farm of the Universidad Autonoma de Aguascalientes, Aguascalientes, Mexico. The farm is used for breeding and fattening pigs for teaching-learning purposes. Random water samples were aseptically obtained from drinkers located on the floor of barnyard from select areas on the farm (26). Water samples were taken directly from the drinkers at its deepest zone with 50 ml sterile Corning tubes. Samples were stored at room temperature until used for bacteria isolation.

### Isolation of bacteria from drinking water samples

Samples were centrifuged at 10,000 × g for 10 min to recover the bacteria and the supernatant was discarded. The obtained pellets were re-suspended in the remaining volume. Dilutions were made in distilled water in the order 10^3^, 10^4^, 10^5^, 10^6^, plated on BHI (agar brain-heart infusion, Bioxon, Mexico) and incubated at 37°C for 24 h. Colonies of each bacterium were plated alone on BHI agar and incubated at 37°C for 24 h. All isolated bacteria were stored in glycerol 30% and stored at −80°C.

### Characterization of isolates

Once isolation of bacteria from drinking water was made, morphological characterization of the colonies and the biochemical tests such as Gram stain, catalase and oxidase were performed. All the strains were evaluated by Api NE biochemistry test (BioMérieux, France), according to the manufacturer's instructions.

### Confirmation of *E. coli* isolation

*Escherichia coli* isolation was confirmed by PCR as previously reported (28) by de presence of *uidA* gene, which encodes the beta-glucuronidase enzyme. Phylogenetic group of each strain was identified (29). *Escherichia coli* isolated from drinking water were screened for the presence of selected virulence genes usually associated with the *E. coli* strains responsible for extra-intestinal infections, including: *fyuA* (yersiniabactin receptor), *kpsMTII* (capsular polysaccharide genes), and *papC* [P fimbriae, (30)]. In order to detect the genes *agn*43 [antigen 43, (31)], *fimH* [minor component of type 1 fimbriae, (32)], *hlyA* [haemolysin, (33)], and *afa* [afimbrial adhesins, (30)] a multiplex PCR was designed with the following conditions: 94°C for 5 min followed by 40 cycles of 30 sec at 94°C, 1 min at 60°C and 1 min at 68°C with a final elongation step at 72°C for 10 min. For the sequences of the primers see Table 1. The amplification products were observed by electrophoresis in 1.5% agarose gel stained with 1 μg ethidium bromide ml^−1^.

**Table 1:** Sequence of primers used for the confirmation of the *E. coli* isolated.

**Gene**.	**Primer Name**.	**Sequence (5^′^-3^′^)**.	**Size (bp)**.	**References**.
*fyuA*	FyuAf FyuAr	TGATTAACCCCGCGACGGGAA CGCAGTAGGCACGATGTTGTA	880	29
*kpsMTII*	KpsMIIf KpsMIIr	GCGCATTTGCTGATACTGTTG CATCCAGACGATAAGCATGAGCA	272	29
*papC*	papC-forward papC-reverse	GACGGCTGTACTGCAGGGTGTGGCG ATATCCTTTCTGCAGGGATGCAATA	350	30
*arpA*	AceK.f ArpA1.r	AACGCTATTCGCCAGCTTGC TCTCCCCATACCGTACGCTA	400	29
*chuA*	chuA.1b chuA.2	ATGGTACCGGACGAACCAAC TGCCGCCAGTACCAAAGACA	288	29
*yjaA*	yjaA.1b yjaA.2b	CAAACGTGAAGTGTCAGGAG AATGCGTTCCTCAACCTGTG	211	29
TspE4.C2	TspE4C2.1b TspE4C2.2b	CACTATTCGTAAGGTCATCC AGTTTATCGCTGCGGGTCGC	152	29
*arpA*	ArpAgpE.f ArpAgpE.r	GATTCCATCTTGTCAAAATATGCC GAAAAGAAAAAGAATTCCCAAGAG	301	29
*trpAgpc*	trpAgpC.1 trpAgpC.2	AGTTTTATGCCCAGTGCGAG TCTGCGCCGGTCACGCCC	219	29
*fimH*	*fimH* F *fimH* R	GGGGGTGCACTCAGGGAACCATTCAGGCA GGGGCATGCTTATTGATAAACAAAAGTCAC	502	32
*agn*43	*agn*43 F *agn*43 R	TTCCGGGAAGACGGTGAA TTCTGGGTGAGTGTGGTGTTG	143	31
*afa*	*afa* F *afa* R	GGCAGAGGGCCGGCAACAGGC GCAATGTACGCGGTCGTTAACGTC	559	30
*hlyA*	*hlyA* F *hlyA* R	AACAAGGATAAGCACTGTTCTGGC ACCATATAAGCGGTCATTCCCGTCA	1176	33

The strains used for positive controls were: *E. coli* strains H10407, E22, CFT073, ECOR 70, 042, EDL933, and ECOR 36. All control strains were kindly provided by Laboratoire de référence pour *Escherichia coli*, EcL, Faculté de Médecine Vétérinaire, Université de Montréal.

### *Escherichia coli* biofilm formation

Mono-species biofilms of *E. coli* isolates were obtained as previously described (34, 35) with modification. Culture medium for the formation of these mono-species biofilm was Luria Bertani (LB). Briefly, overnight cultures of *E. coli* were diluted 1/100 in LB broth plus glycerol (0.20%). A volume (100 μl) was aliquoted by triplicate in wells of a sterile 96-well microtiter plate (Costar® 3599, Corning, NY, USA). *E. coli* strains ATCC 25922 (clinical isolate [American Type Culture Collection, Manassas, VA, US]) and L17608 (swine isolate) were used as positive controls. Wells containing sterile broth were used as negative controls. Following an incubation of 24 h at 37°C, the plate was washed by immersion in water. Biofilms were then stained with 0.1% (w/v) crystal violet for 2 min, rinsed once with distilled water, dried at 37°C for 30 min, and then, 100 μl of ethanol (70%) were added to the wells. Absorbance was measured at 590 nm using a spectrophotometer. For the qualitative determination of the ability of *E. coli* to form biofilm, the previously described methodology was carried out (36).

### Integration of *actinobacillus pleuropneumoniae* in biofilms formed by *escherichia coli*

To analyze the incorporation of *A. pleuropneumoniae* in preformed *E. coli* biofilms, a previously described methodology with several modifications was used (27). For this test, two strains of *A. pleuropneumoniae* (reference strain 4074 and swine isolated strain 719), both belonging to serotype 1 and biotype 1, were used. Briefly, overnight cultures of *A. pleuropneumoniae* grown in BHI broth plus NAD (15 μg/ml) and *E. coli* grown in LB culture media, were diluted 1/100 in LB broth plus glycerol (0.20%). A volume (200 μl) was aliquoted by triplicate in wells of a sterile 96-well microtiter plate (Costar® 3599, Corning, NY, USA) using the following template: 100 μl *A. pleuropneumoniae* in BHI (glycerol 0.20%) plus 100 μl *E. coli* in LB (glycerol 0.20%) and incubated 24 h at 37°C. Wells containing sterile broth or *A. pleuropneumoniae* (100 μl of bacteria plus 100 μl of LB-glycerol 0.20%) were used as blank and negative control, respectively (*A. pleuropneumoniae* it is unable to grow and form biofilms under these conditions). Wells containing *E. coli* ATCC 25922 and L17608 (100 μl of bacteria plus 100 μl of LB-glycerol 0.20%) were used as positive controls for biofilm formation.

### Colony forming unit (CFU) counts of mono and two-species biofilms

To confirm the presence of *A. pleuropneumoniae* and *E. coli* in the biofilms, the colony forming units (CFU) were counted, using selective growth media and colony morphology. The CFU test was performed as previously described (27, 37) with modifications. Briefly, the medium was carefully removed from each well by pipetting and washed with 200 μl of sterile water. Twenty microliters of NaCl 0.85% were added. A tip was used to scrape the bottom and completely disintegrate the biofilm, taking 20 μl to perform serial dilutions in saline solution 0.85% (from 10^−2^ to 10^−7^). Finally, 100 μl of the dilution were plated on BHI, BHI plus NAD, and Blood agar plus NAD (*A. pleuropneumoniae* causes beta-hemolysis), incubated 24 h at 37°C, and the CFU count was performed.

### Confocal laser scanning microscopy (CLSM)

In order to study the morphology of mono and two-species biofilms, *E. coli* biofilms with or without *A. pleuropneumoniae* 719 were prepared as described above and stained with FilmTracer FM 1-43 (Invitrogen, Eugene, OR), Wheat Germ Agglutinin (WGA-Oregon Green 488, Molecular Probes), Film Tracer TM SYPRO® Ruby biofilm matrix stain (Molecular Probes), or BOBOTM-3 iodide (Molecular Probes) according to manufacturer's instructions (fluorescent markers stain bacterial membranes, N-acetyl-Dglucosamine [PGA] and N-acetylneuraminic acid residues, proteins and extracellular DNA or eDNA, respectively). After 30 min of incubation at room temperature, the fluorescent marker solution was removed, and the biofilms were washed with water. After that, the biofilms were observed by confocal laser scanning microscopy (CLSM; LMS 700 ZEISS; Carl Ziess Microscopy, Jena, Germany) and images were acquired using Zen Black 2012 (black edition) software (ZEISS).

### Enzymatic treatments of two-species biofilms

The enzymatic treatment assays were performed as described previously (3) for proteinase K and DNase I, and (38) for cellulase. Biofilms were prepared as described above and 50 μL of proteinase K (500 μg/mL in 50 mM Tris-HCl pH 7.5, 1 mM CaCl_2_), 50 μL of DNase I (500 μg/mL in 150 mM NaCl, 1 mM CaCl_2_), or 50 μL of cellulase (40 μU/ml in 100 mM C_2_H_3_NaO_2_, 50% DMSO) were added directly to the biofilms. Samples with proteinase K or DNase I were incubated for 1 h at 37°C, and with cellulose were incubated 30 min at 37°C. Control wells were treated with 50 μL of the buffer without the enzyme. Biofilms were washed and stained with crystal violet and the absorbance was measured at 590 nm.

### Phenotype assay: congo-red and calcofluor

Congo-red and calcofluor assays were performed in order to determine the production of fimbriae-curli and cellulose, and were performed as described previously (38). For the assay, a 2 μl drop of bacterial culture was taken from liquid medium, and was placed on Luria-Bertani salt-free plates (LB; Difco Laboratories, Detroit, MI), containing 0.02% of Congo-red (Sigma®, C-6767) and 0.002% of Coomassie brilliant blue G (Sigma®, F3546-5G) for fimbriae detection, and containing 0.02% calcofluor (fluorescent brightener 28, Sigma-Aldrich® F-3543) dissolved in 1 mM HEPES for cellulose detection. For the two-species assay, a 1:1 dilution of the bacterial cultures (*E. coli* plus *A. pleuropneumoniae*) was performed in respective culture media. Twelve strains were placed per plate with a centimeter of distance between each drop. After seeding, the plates were allowed to dry for 5 to 10 min face-up and then incubated for 48 hat 30°C. The fluorescence of the colonies was verified by UV light illumination (360 nm) after overnight incubation at 30°C. *E. coli* CFT073 and *E. coli* ATCC 25922 were used as positive and negative controls, respectively (39).

### Scanning electron microscopy

The two-species biofilm formed by *A. pleuropneumoniae* 719-*E. coli* ATCC 25922 was observed under electron microscopy (SEM). Mono-species biofilms of *A. pleuropneumoniae* and *E. coli* were used as positive controls and were grown as described previously. The two-species biofilm was prepared as described above. Samples were processed as described Loera-Muro et al. (26), and were observed with a Jeol LV-5900 scanning electron microscope. The bacteria dimensions were measured with the microscope software. The experiment was repeated three times, measured between 3 and 5 bacteria in three different fields.

### Statistical analysis

Statistical significance analyses (*p*-value < 0.05) of differences in biofilms were determined by Two-way ANOVA followed by Tukey's test using GraphPad Prism version 4.0 (GraphPad Software, San Diego, CA, USA).

## Results

### Identification of bacteria from drinking water of swine farms

A total of 10 samples of drinking water from pig farm were obtained. After performing the isolation of bacteria from water, 52 colonies were selected for identification, 63.46% (33/52) of them were Gram-negative and 36.54% (19/52) were Gram-positive. Gram-negative bacteria belonged to the following species: *Escherichia coli* (30.30%, 10/33)*, Enterobacter cloacae* (3.03%, 1/33)*, Pseudomonas aeruginosa* (9.09%, 3/33), *P. fluorescens* (9.09%, 3/33)*, Photobacterium damselae* (3.03%, 1/33)*, Salmonella* spp. (12.12%, 4/33)*, Ochrobactrum anthropic* (9.09%, 3/33)*, Pasteurella pneumotropica* (3.03%, 1/33)*, Cryseumonas luteola* (3.03%, 1/33)*, Kluyvera* spp. (6.06%, 2/33)*, Citrobacter freuindii* (6.06%, 2/33)*, Buttiauxella agrestis* (3.03%, 1/33), and *Cedecea lapagei* (3.03%, 1/33). Bacteria identified as *E. coli* were selected for characterization of two-species biofilm formation with *A. pleuropneumoniae* since *E. coli* was the main species in the samples.

### Characterization of the *E. coli* isolates

Ten *E. coli* isolates were characterized by PCR. All isolates belonged to the group of extra-intestinal pathogenic *E. coli* [strains carrying ExPEC related genes: *fyuA, papC, kpsMTII, afa, fimH, agn43*, and *hlyA* (30–33)]. Phylogroups detected include: D (40%, 4/10), B1 (20%, 2/10), A (20%, 2/10), C and E (10%, 1/10) groups (Table 2).

**Table 2:** Characterization of *E. coli* isolated from drinking water of swine farm.

	**Virulence factors**								**Phylogroup**						
**Isolated**	***fyuA***	***kpsMTII***	***papC***	***fimH***	***agn*****43**	***afa***	***hlyA***	**Group**	***arpA***	***chuA***	***yjaA***	**TspE4.C2**	***arpA*** **(group E)**	***trpA*** **(group C)**	**Phylogroup**
8–1	+	+	+	–	–	–	–	ExPEC^*^	+	+	–	–	–	NA	D
8–2	+	+	+	–	–	–	–	ExPEC^*^	+	+	–	–	–	NA	D
8–3	+	+	+	–	–	–	–	ExPEC^*^	+	+	–	–	–	NA	D
8–4	+	+	+	–	–	–	–	ExPEC^*^	+	+	–	–	–	NA	D
13–1	+	+	+	+	–	–	–	ExPEC^*^	+	-	–	+	NA	NA	B1
13–2	+	+	+	+	–	–	–	ExPEC^*^	+	-	–	+	NA	NA	B1
13–5	+	+	+	+	+	–	–	ExPEC^*^	+	-	+	–	NA	+	C
14–1	+	+	+	+	+	–	–	ExPEC^*^	+	–	–	–	NA	NA	A
14–2	+	+	+	+	+	–	–	ExPEC^*^	+	–	–	–	NA	NA	A
14–5	+	+	+	–	+	–	–	ExPEC^*^	+	+	–	–	+	NA	E

+, Positive sample; -, Negative sample; NA, not searched with primers specific for the phylo groups C and D;

* *,strains carrying ExPEC related genes*.

### *Escherichia coli* biofilm formation and integration of *A. pleuropneumonia* into biofilms

**Figure 1:**
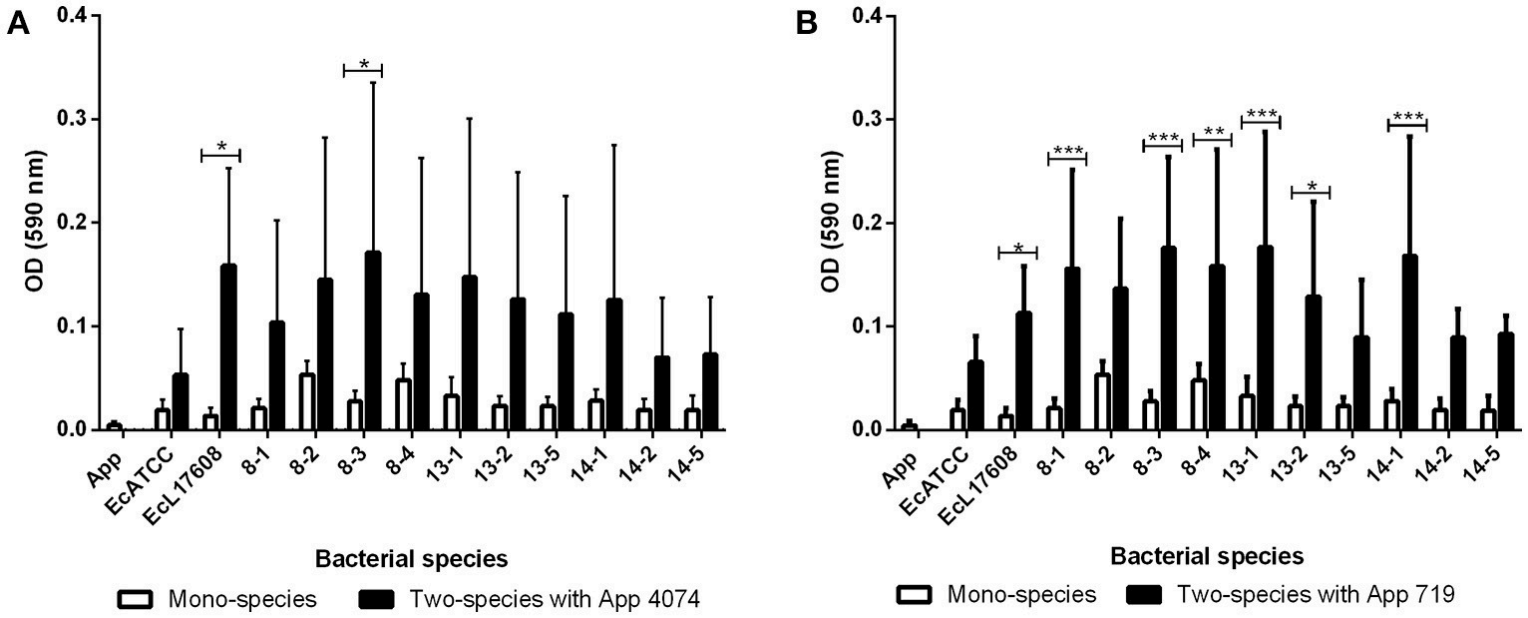
Mono-species and two-species biofilms formed by *E. coli* isolates from drinking water of a swine farm alone or with *A. pleuropneumoniae*. **(A)** Mono-species biofilms of control *A. pleuropneumoniae* 4074 and each *E. coli* isolate and two-species biofilms formed with *A. pleuropneumoniae* 4074 and each *E. coli* isolate. **(B)** Mono-species biofilms of control *A. pleuropneumoniae* 719 and each *E. coli* isolate, and two-species biofilms formed with *A. pleuropneumoniae* 719 and each *E. coli* isolate. All the statistically significant differences in the production between mono-species and two-species biofilms are pointed. ^*^p < 0.05, ^**^p < 0.01, ^***^p < 0.001, ^****^p < 0.0001.

Mono-species biofilm formation of 10 *E. coli* isolates and two controls, belonging to the strains ATCC 25922 and L17608 were performed (Figure 1). Integration of *A. pleuropneumoniae* (4074 and 719 strains) in biofilms formed by *E. coli* was also tested. For *A. pleuropneumoniae* 4074, statistically significant (*p* < 0.05; Figure 1A) increments of biofilm formation were detected among the isolates EcL17608 and 8–3. In the case of *A. pleuropneumoniae* 719, 58 percent of the *E. coli* strains (7/12 strains), showed statistically significant *p* < 0.05; Figure 1B) increments in biofilm formation.

**Figure 2:**
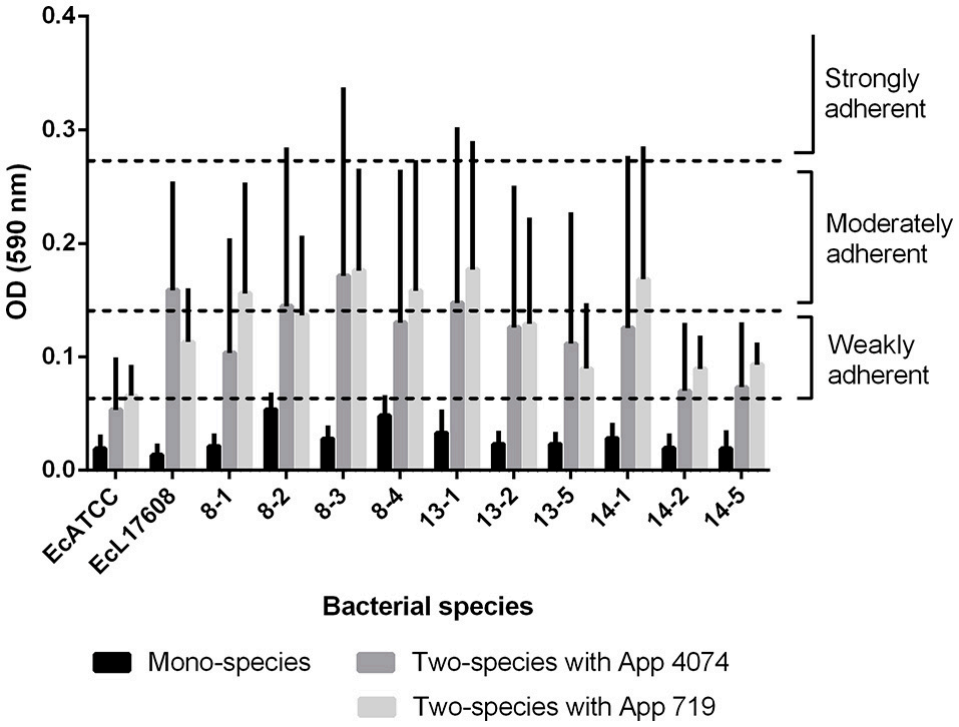
Classification of biofilm formation capacity as weak, moderate and strongly adherent in *E. coli* mono-species biofilms and *E. coli*- *A. pleuropneumoniae* two-species biofilms (*E. coli*- *A. pleuropneumoniae* 4074 and *E. coli*- *A. pleuropneumoniae* 719). In all cases it can be observed that addition of *A. pleuropneumoniae* promotes increases on biofilm production, from weak when are mono-species to moderate when are two-species.

Moreover, based on the results of the assay for two-species biofilm formation, the methodology of (36) was applied for the qualitative determination of the ability to form biofilm. The optical density of control (ODc) was defined as the mean OD of the negative control and strains were classified as non-adherent (OD ≤ ODc), weakly adherent (ODc < OD ≤ 2 × ODc), moderately adherent (2 × ODc < OD ≤ 4 × ODc), or strongly adherent (OD > 4 × ODc). In the mono-species biofilms, only 8–2 and 8–4 *E. coli* isolates were able to form a weak adherent biofilm (Figure 2). However, biofilm formed by *A. pleuropneumoniae* 4074 or 719 with *E. coli* increased biofilm formation from weakly adherent to moderately adherent in some combinations (Figure 2). In the case of two-species biofilms with *A. pleuropneumoniae* 4074, six isolates increased their adhesion ability to weakly adherent (8–1, 13–2, 13–5, 14–1, 14–2, and 14–5 isolates) and the remaining isolates increased their adhesion ability to moderately adherent (8–2, 8–3, 13–1 isolates and the control Ec L17608). The control ATCC 25922 did not suffer any modification (Figure 2). For *A. pleuropneumoniae* 719, six of the isolates increased their adhesion ability to moderately adherent (8–1, 8–2, 8–3, 8–4, 13–1, and 14–1) and the remaining six isolates including the controls ATCC 25922 and Ec L17608 increased their adhesion ability to weakly adherent (13–2, 13–5, 14–2, and 14–5 isolates).

### Colony forming unit counts of mono and two-species biofilms

**Figure 3:**
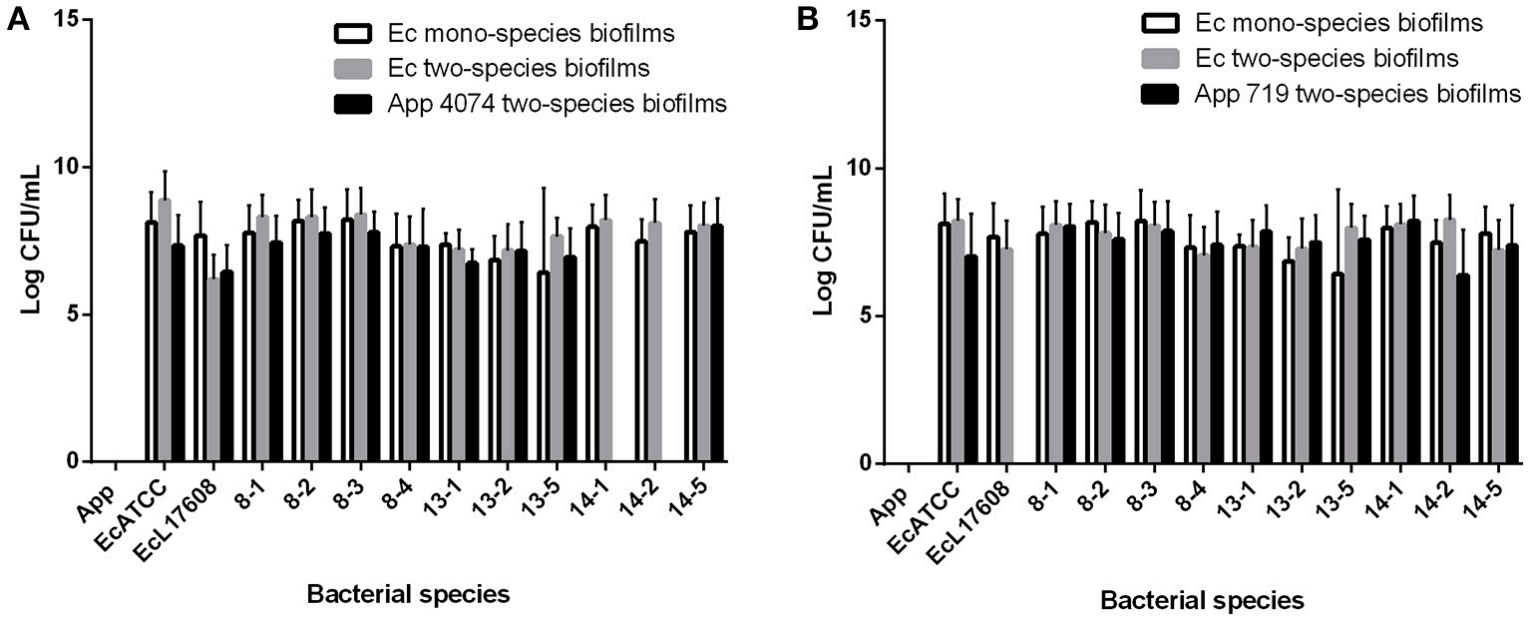
Colony forming units (CFU) counts from mono and two-species biofilms formed by *A. pleuropneumoniae* with the *E. coli* isolates from drinking water of a swine farm. **(A)** CFU of two-species biofilms formed by *A. pleuropneumoniae* 4074 with each *E. coli* isolate. **(B)** CFU of two-species biofilms formed by *A. pleuropneumoniae* 719 with each *E. coli* isolate. Ec mono-species biofilms bar represent the CFU of *E. coli* bacteria from the mono-species biofilms. Ec two-species biofilms and App two-species biofilms bar represent the CFU of each bacterium from the two-species biofilms.

In the case of the CFU count, the numbers of *A. pleuropneumoniae* and *E. coli* found in two-species biofilms were similar in almost all cases (Figure 3). No significant differences between populations were shown. These results suggest strongly that *A. pleuropneumoniae* has the ability to be incorporated into biofilms produced by environmental bacteria, which supports that *A. pleuropneumoniae* is using the multi-species biofilms as a survival strategy in the environment, at least for 72 h of interaction. For all subsequent assays, *A. pleuropneumoniae* reference strain 719 was selected because this strain isolated from pigs has a high capacity for biofilm formation.

### Two-species biofilms matrix composition

**Figure 4:**
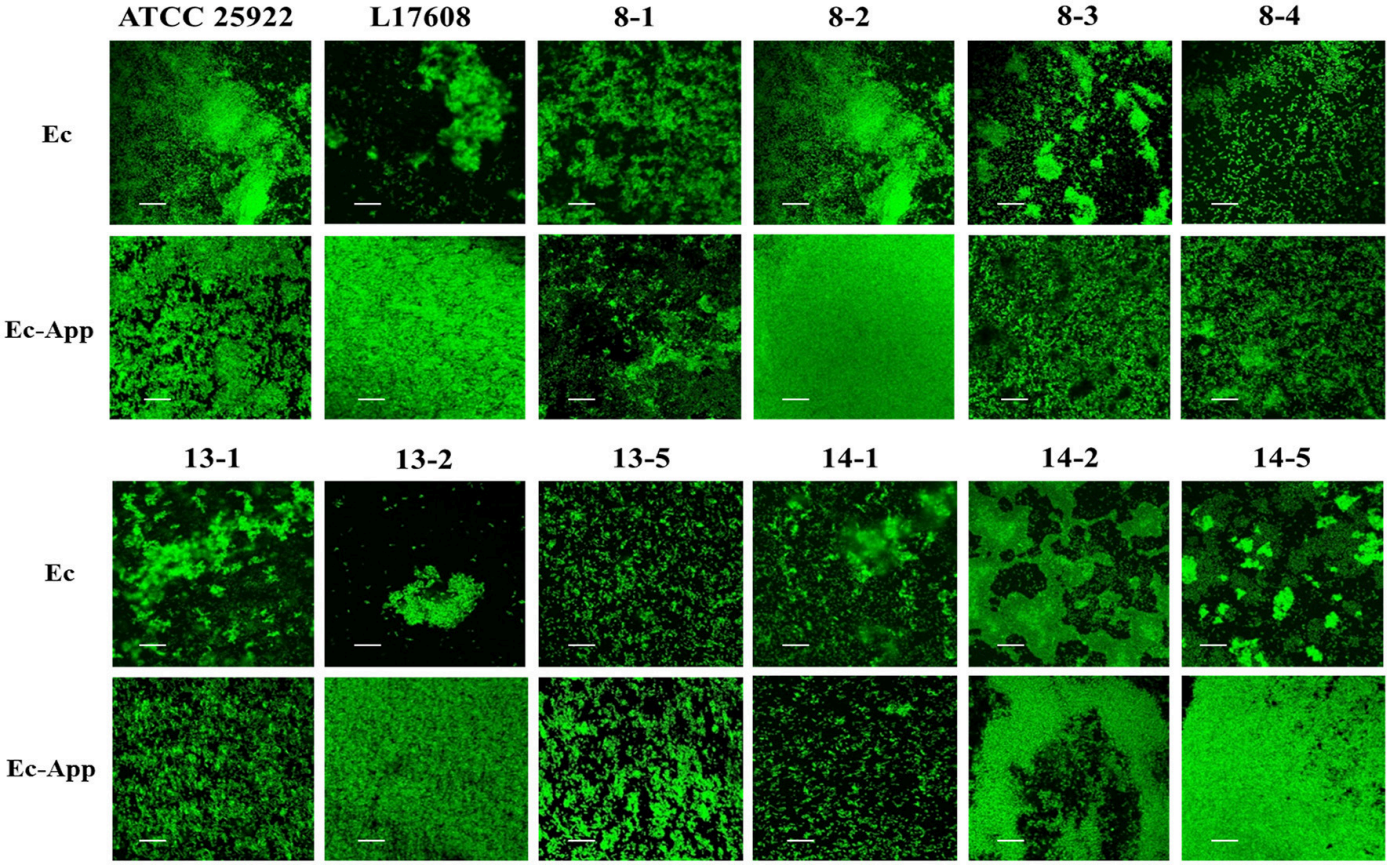
*Escherichia coli* strains in mono- or two-species biofilms with *A. pleuropneumoniae* (strain 719) observed by confocal laser scanning microscopy. Biofilms were stained with FM-143. The panel shows views from the top of biofilms. Ec; *E. coli*, App; *A. pleuropneumoniae* 719. Scale bar 30 μm.

**Figure 5:**
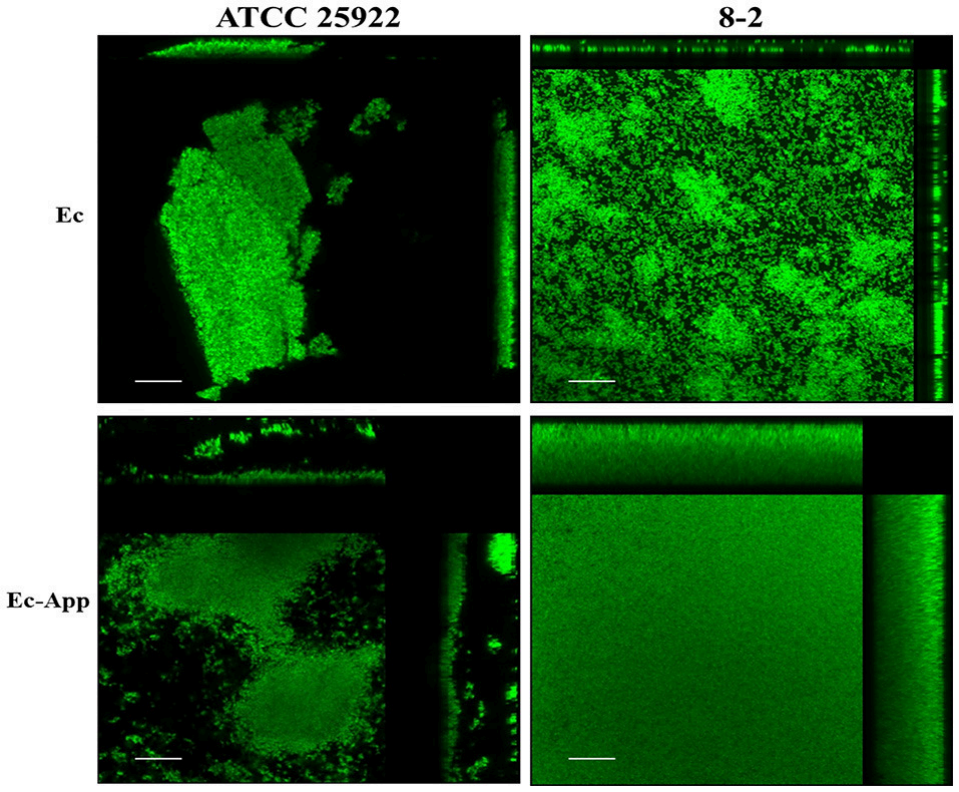
Confocal laser scanning of mono or two-species biofilms formed by *E. coli* alone or with *A. pleuropneumoniae*. Images show the increment in the biofilms when the bacteria are in multi-species (stained with FM-143). Side panels (top and right) show cross-section views from the sides of the biofilms Ec*; E. coli*, App; *A. pleuropneumoniae* 719. Scale bar 30 μm.

**Figure 6:**
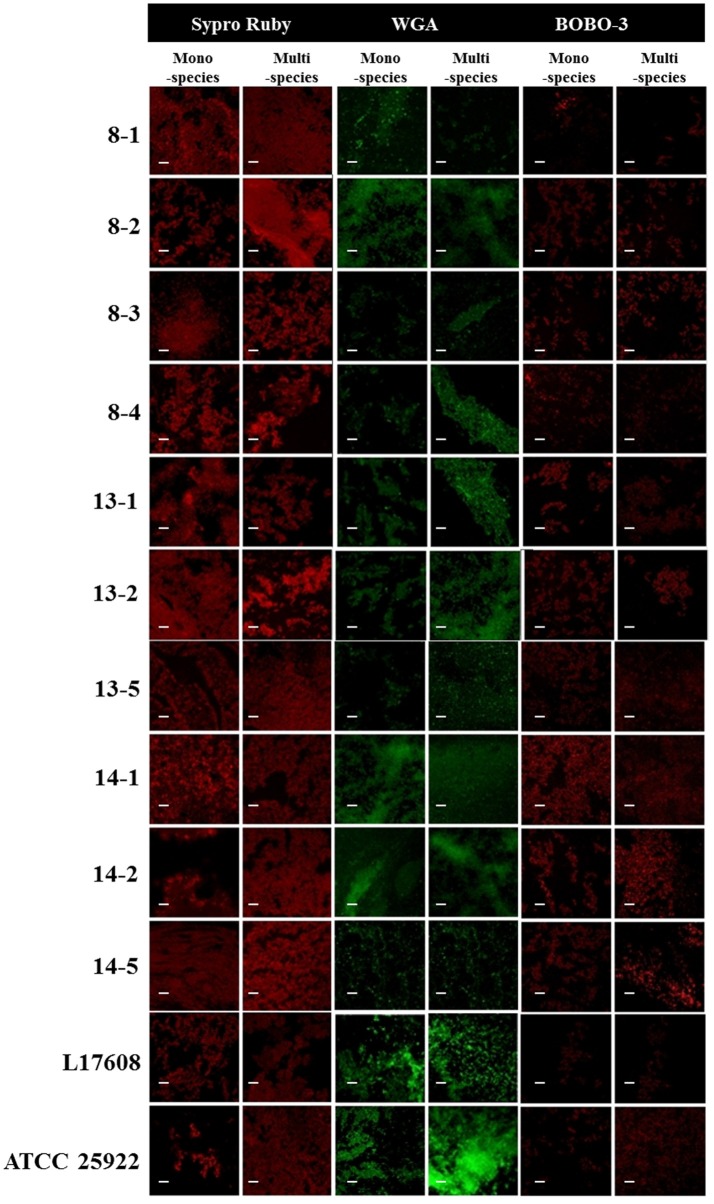
*Escherichia coli* strains in mono- or two-species biofilms with *A. pleuropneumoniae* (strain 719) observed by confocal laser scanning microscopy. Images show the mono-species biofilms of *E. coli* isolates and two-species biofilms of *E. coli* isolates and *A. pleuropneumoniae* 719 in LB media stained with wheat-germ agglutinin (WGA)-Oregon green, SYPRO Ruby, and BOBO-3 (all from Invitrogen, Eugene, OR). Scale bar 30 μm.

Confocal laser scanning microscopy (CLSM) and the dye FM-143 was carried out to visualize biofilm morphology from both, mono- and two-species biofilm. Observation of several fields on each sample evidenced by increments in most of the two-species biofilms formed with different *E. coli* strains and the *A. pleuropneumoniae* isolates, as compared to the mono-species biofilms of *E. coli* (Figure 4). As a whole, these images are in accordance with the results obtained by the crystal violet technique. Likewise, it was observed that the biofilms morphology had some changes (Figures 4, 5). Otherwise, biofilm matrix components were characterized by CLSM in combination with different dyes directed mainly toward PGA, eDNA, and proteins. These three macromolecules were detected in the extracellular matrix (Figure 6). In some cases, the production of proteins, PGA and eDNA, stained with SYPRO Ruby, WGA, and BOBO-3; respectively, showed an increase in biofilms formed by two-species compared to mono-species biofilms (Figure 6). The data obtained from the SYPRO Ruby stain, which labels most classes of proteins, showed a protein increase in the two-species biofilms *E. coli* 14–2 and EcATCC with *A. pleuropneumoniae* 719. The isolates 8–4, 13–1, 13–2, 13–5, 14–1, and EcATCC were stained with WGA, suggesting the increase in the presence of PGA or at least in the presence of *N*-acetyl-d-glucosamine and N-acetylneuraminic acid residues in the biofilm matrix. BOBO-3 iodide that stains extracellular DNA showed an increase only among the isolates 14-2 and EcATCC.

**Figure 7:**
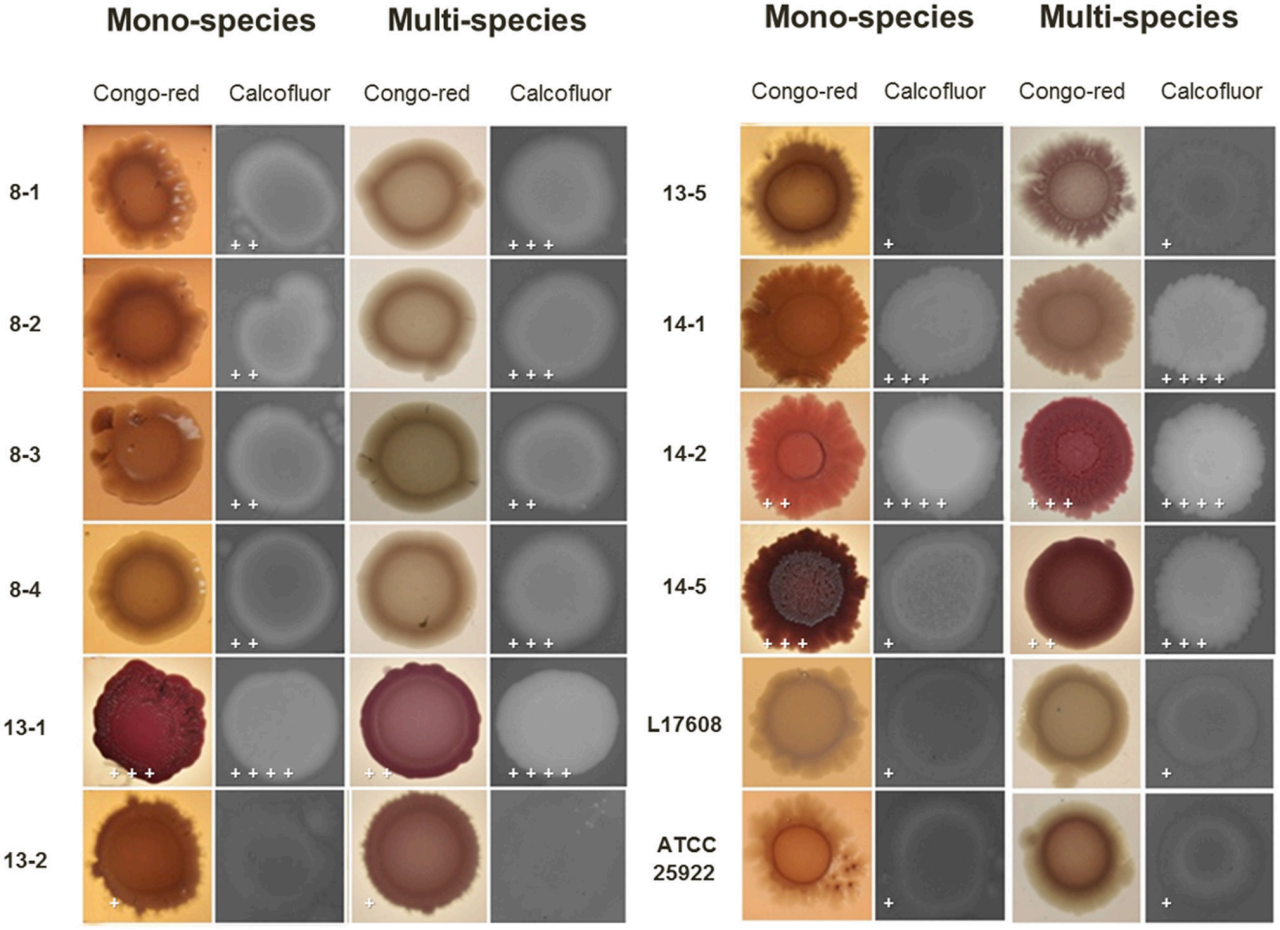
Congo-red and calcofluor binding assays on mono or two-species colonies formed by *E. coli* alone or with *A. pleuropneumoniae*. Congo-red assay evidenced curli-producing bacteria when *E. coli* was growing on congo-red-supplemented nutrient agar. Moreover, changes in cellulose production were detected by the calcofluor assay. The plus sings indicate differences on production observed in the colonies of one or two-species. The crosses indicate the qualitative production of cellulose seen in each of the biofilms (+ low, ++ medium, +++ medium high and ++++ high).

Congo-red has been used extensively to supplement nutrient agar to distinguish the production of the extracellular matrix components cellulose and curli fimbriae from non-cellulose curliated bacteria. Likewise, the phenotype on calcofluor plates served as an indicator of cellulose production. In this work, the presence of few fimbriae-curli forming, and cellulose producer strains was observed (13-1, 13-2, 13-5, 14-1, 14-2, and 14-5, Figure 7). Also, changes were observed when the *E. coli* strains were together to *A. pleuropneumoniae* in the cellulose and curli production. These results confirm changes in the production of extracellular matrix components in two-species biofilms as compared to the *E. coli* mono-species.

**Figure 8:**
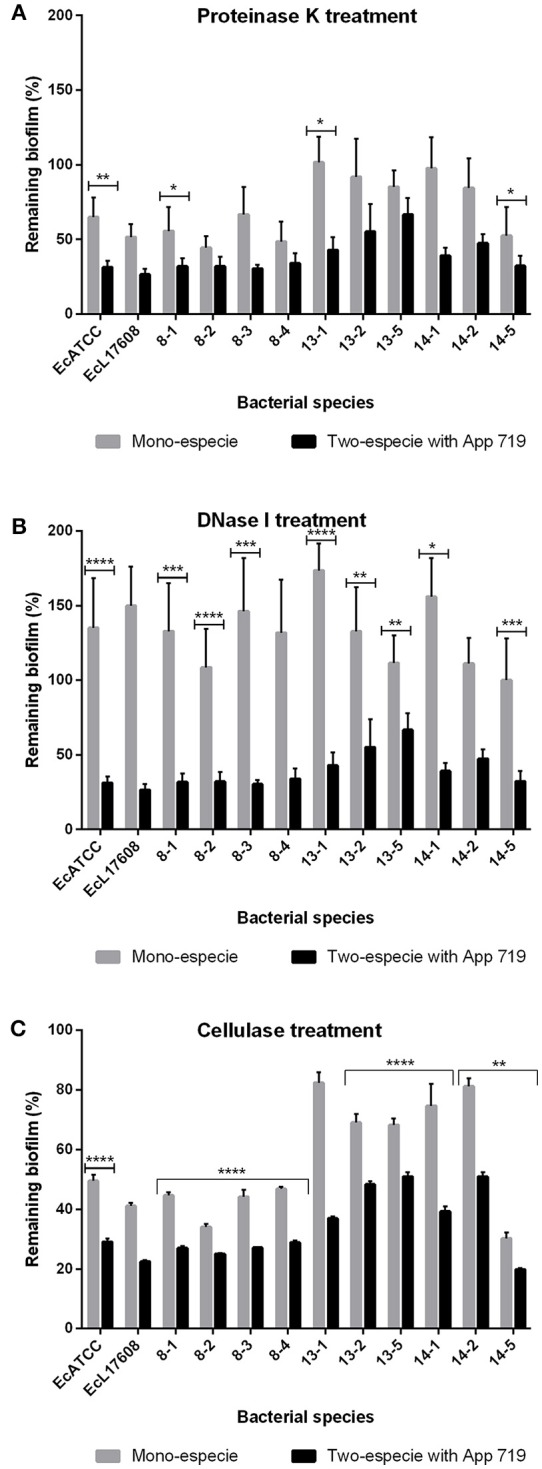
Mono or two-species biofilms of *E. coli* and *A. pleuropneumoniae* enzymatic treatment. Dispersion by **(A)** proteinase K, **(B)** DNase I, and **(C)** cellulase of mono or two-species biofilms formed by *E. coli* strains alone or with *A. pleuropneumoniae* strain 719 grown in LB media. ^*^p < 0.05, ^**^p < 0.01, ^***^p < 0.001, ^****^p < 0.0001.

To determine the structural roles played by the compounds forming the extracellular matrix, enzymatic treatments were performed on the two and mono-species biofilms (Figure 8). Treatment with proteinase K, DNase I and cellulose provoked reduction in all two-species biofilms. It was more important that the effect observed in the mono-species biofilms. These results indicate that when biofilms of two-species are being formed, the cellulose, as well as proteins and eDNA, take a structural function as occur in mono-species biofilms.

### Scanning electron microscopy

**Figure 9:**
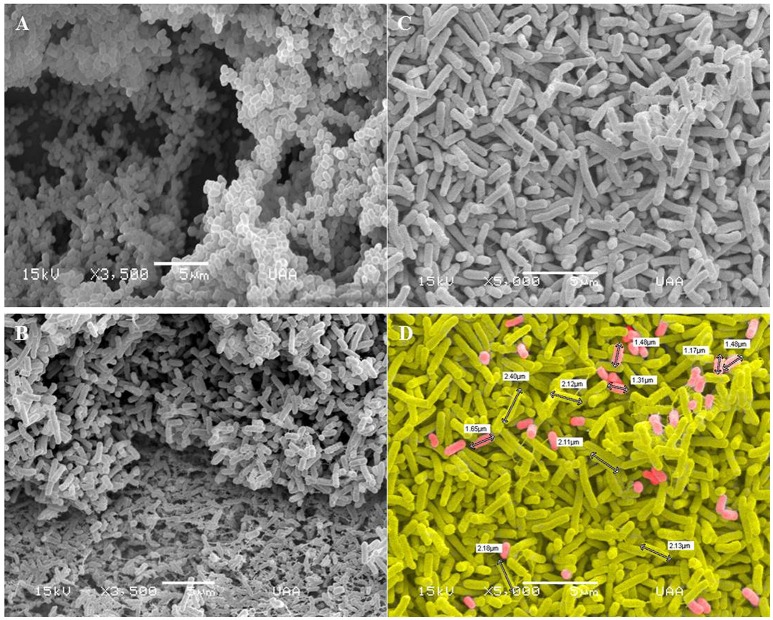
Scanning electron microscopy images of biofilms formed by *A. pleuropneumoniae* and *E. coli*. Mono-species and two-species biofilms constituted by **(A)** *A. pleuropneumoniae* (719), **(B)** *E. coli* (ATCC 25922) (Magnification: 3500x) and **(C–D)** *A. pleuropneumoniae* and *E. coli* (719 and ATCC 25922 respectively) (Magnification: 5000x). Fimbria- and curli-like structures in biofilms formed by *E. coli* and *E. coli*- *A. pleuropneumoniae* are indicated. **(D)** shows the sizes of bacteria (white labels); cells were painted to distinguish the two apparent populations of bacteria. Scale bar 5 μm.

*Actinobacillus pleuropneumoniae* and *E. coli* two-species biofilms were analyzed by SEM (Figure 9). It was possible to observe the presence of two populations in the two-species biofilms, an abundant population of larger bacteria, and a minor population of smaller bacteria (*p* < 0.01, Figure 9D). It was also interesting to observe fimbriae-like or curli-like structures, and their promotion of interaction between all the bacteria present in the biofilm. Moreover, these structures appear more abundant in the *E. coli* mono-species biofilm (Figure 9B) than in the *E. coli*—*A. pleuropneumoniae* two-species biofilm (Figure 9C).

## Discussion

Porcine pleuropneumonia caused by *A. pleuropneumoniae*, is one of the most important porcine respiratory diseases which is spread by direct contact between the carrier-infected pig and an uninfected pig or by aerosols (1–5). The indirect route of transmission via surface was not considered very important and therefore the ability of *A. pleuropneumoniae* to survive in the environment outside of its host is not yet known (25). Previous studies from our group demonstrated that *A. pleuropneumoniae* is able to grow in unsuitable environments when forming multi-species biofilms with other respiratory pathogens of pigs that are also part of the porcine respiratory disease complex (6, 17, 27). In this study, a total of 10 samples of drinking water were taken from a swine farm in the State of Aguascalientes where previously Loera-Muro et al. (26) detected the presence of *A. pleuropneumoniae* in drinking water using a specific PCR for *apxIV* gene. We found that some microorganisms that form the microbial community of drinking water of swine farms were bacteria such as *Escherichia, Enterobacter, Pseudomonas, Photobacterium, Salmonella, Ochrobactrum, Pasteurella, Cryseumonas, Kluyvera, Citrobacter*, and *Buttiauxella*. The more abundant culturable bacterium isolated from samples of drinking water from a swine farm in the State of Aguascalientes was *E. coli*. All *E. coli* isolates belonged to the group of extra-intestinal pathogens (ExPEC). ExPEC are facultative pathogens, which can reside in the gastrointestinal tract of a certain fraction of the human and animal population. They possess several virulence traits that allow them to colonize different niches including urogenital tract resulting in urinary tract infections (UTIs), meningitis and sepsis in animals and humans (40). In pigs, these pathogens could cause fatal pneumonia, severe septicaemia and haemorrhagia; thus, they also represent a latent risk for human health (41–43). The presence of ExPEC strains may indicate a zoonotic potential risk posed by swine farms to cause infections by ExPEC stains in both, pigs and humans, mainly farm workers.

To seek out interactions during biofilm formation in two-species biofilms between the swine respiratory pathogen *A. pleuropneumoniae* (strain 4074 and 719), and *E. coli* isolated from drinking water, different approaches were undertaken. An increase in biofilm formation was evidenced by crystal violet staining, when two-species biofilms were compared to those obtained with the *E. coli* mono-species assay. This result suggests an interaction between both bacteria affecting bacterial distribution and probably biomass production as reported by others (44). Furthermore, by applying the methodology of (36), for the qualitative determination of *E. coli's* ability to form biofilm, this increment was observed. When *A. pleuropneumoniae* was combined with any of the *E. coli* strains, the biofilm classification changed from non-adherent to weakly adherent or moderately adherent. In addition, we were able to recover *A. pleuropneumoniae* from the biofilm in most cases, which was unexpected considering biofilm was cultured in optimal conditions for *E. coli* growth, but not for *A. pleuropneumoniae*. Thus, the ability of *A. pleuropneumoniae* to form two-species biofilm with *E. coli* isolated from drinking water was confirmed. This interaction occurs because *E. coli* possibly supplies some nutrients that promote *A. pleuropneumoniae* growth (27).

There are several reports on the advantages obtained during multi-species biofilm formation. Liu et al. (45) determined the capacity to incorporate the bacteria *E*. *coli* O157:H7 in pre-formed biofilms with bacteria obtained during the fresh produce processing environments. When co-cultured with *E. coli* O157:H7, *Burkholderia caryophylli*, and *Ralstonia insidiosa* exhibited increases in biofilm biomass, which were around 180 and 63%, respectively; as well as in the thickness of the biofilm. Biyikoglu et al. (46) reported that *Actinomyces oris* and *Veillonella parvula* promoted biofilm growth of all *Fusarium nucleatum* strains tested in their study. Both studies reported similar effects: increases in biofilms when they are formed by multiple species, such as in our case. The results presented here are in accordance with the study carried out by Bridier et al. (47), where pathogenic *Staphylococcus aureus* grown in mixed biofilm with the *Bacillus subtilis* ND medical strain, was protected from peracetic acid (PAA), an oxidizing agent, thus enabling its persistence in the environment. Standar et al. (37) also showed that two-species combinations of *Streptococcus mitis* with either *Streptococcus mutans* or *Aggregatibacter actinomycetemcomitans* favored bacterial interactions influencing biofilm mass, biofilm structure and cell viability. The result reported by Standar et al. (37) is similar to that observed in our study where *E. coli* in presence of *A. pleuropneumoniae* favored an increase in biofilm formation, allowing it to survive even under conditions unfit for its development. Likewise, the integration of pathogenic bacteria in biofilms formed by other bacteria was shown by Stewart et al. (48), where *Legionella pneumophila* 130b persisted within a two-species biofilm formed by *Klebsiella pneumoniae* and *Flavobacterium* sp., or by *K. pneumonia*, and *P. aeuroginosa*. Furthermore, the authors reported that *Legionella pneumophila* 130b was able to colonize biofilms formed by single-species such as *K. pneumoniae* and *Pseudomonas fluorescens*, and persist in the environment. Finally, (49) using the chinchilla otitis media model concluded that the biofilm formation and persistence on the middle-ear mucosal surface by pneumococcal is facilitated by *Haemophillus influenzae* coinfection. In this study, *A. pleuropneumoniae* was able to colonize and incorporate into biofilms formed by *E. coli*, which might allow it survive in hostile conditions, outside of its host, persisting in the environment as a source for transmission to other pigs.

Considering our results, an interaction is evidenced, between bacteria, *A. pleuropneumoniae* and the *E. coli* environmental isolates. It is unknown whether the increase in biofilm produced when going from mono to two-species is due to the incorporation of *A. pleuropneumoniae* into these biofilms, or a more complex interaction is, causing *E. coli* to over-produce biofilm, via the generation of extracellular matrix components, like cellulose, curli, antigen 43, DNA, β-1,6-N-acetylglucosamine (β-1,6-GlcNAc), capsule sugars, and colanic acid (50, 51). Our working model considers that *A. pleuropneumoniae* is incorporated into *E. coli* biofilms, thus in order to survive and grow in this hostile environment, at least for 72 h, it promotes an increment in biofilm, followed by interactions between both bacteria, resulting in the final increment seen in the two-species biofilms. Moreover, it was also possible to observe that the components of the extracellular matrix in the two-species biofilms changed their function, promoting greater structural stability to the biofilm. In the enzymatic assays a decrease in the biofilm formed by *E. coli* and *A. pleuropneumoniae* was seen, when compared to the biofilms formed only by *E. coli* strains. This change in the structural function of components in the extracellular matrix when going from mono-species to multi-species biofilm had already been reported by our group previously with *A. pleuropneumoniae* (27). However, little is known with regards to other bacterial species (52, 53).

In conclusion, our data suggests that *A. pleuropneumoniae* has the ability to integrate and form multi-species biofilms with environmental bacteria, which could allow it to survive outside of the host, specifically in water, establishing relationships with bacteria from the microbial community of water such as *E. coli*; therefore suggesting a possible mechanism for porcine pleuropneumonia persistence or transmission.

## Author contributions

FR-C directed the biofilms experiments with *Escherichia coli*, mono species, and di-species. AL-M advised the biofilm experiments with *Actinobacillus pleuropneumoniae*, mono species, and di-species. NV-P, CB-G, and AM-F conducted the experiments with biofilms for both species, and they analyzed them by confocal microscopy. FA-G, JH, MJ, and RO advised the management of bacterial strains. F-AG also advised the microbiological analysis. AG-B proposed the research line, is the responsible of the project that support this work, directed five thesis involved in the work.

### Conflict of interest statement

The authors declare that the research was conducted in the absence of any commercial or financial relationships that could be construed as a potential conflict of interest.
